# Seric Molecular Markers Correlated with Stroke Rehabilitation Outcomes: A Narrative Review

**DOI:** 10.3390/life16010183

**Published:** 2026-01-22

**Authors:** Bianca-Gabriela Ene, Brindusa Ilinca Mitoiu, Mariana Catalina Ciornei, Madalina Coman-Stanemir, Angelo Voicu, Floris Petru Iliuta, Ioana Raluca Papacocea

**Affiliations:** 1 Doctoral School, Carol Davila University of Medicine and Pharmacy, 020021 Bucharest, Romania; 2 “Prof. Dr. Agrippa Ionescu” Clinical Emergency Hospital, 011356 Bucharest, Romania; 3 Clinical Department 9, Carol Davila University of Medicine and Pharmacy, 050474 Bucharest, Romania; 4 Discipline of Physiology, Faculty of Medicine, Carol Davila University of Medicine and Pharmacy, 020021 Bucharest, Romania; 5 Department of Psychiatry and Psychology, Discipline of Psychiatry, Faculty of Dental Medicine, Carol Davila University of Medicine and Pharmacy, 010221 Bucharest, Romania; 6 Department of Psychiatry, “Prof. Dr. Alexandru Obregia” Clinical Hospital of Psychiatry, 041914 Bucharest, Romania

**Keywords:** stroke, rehabilitation outcomes, neuroinflammation, neuroplasticity, serum biomarkers, prognostic indicators

## Abstract

An increasing number of stroke survivors are burdened by persistent disabilities, requiring long-term rehabilitation. However, the extent of functional gain is highly variable, severely impairing patients’ quality of life. This variability highlights a critical gap in current prognostic tools, which rely primarily on clinical and neuroimaging data. The aim of this review is to synthesize the current literature on serum biomarkers in stroke survivors and to evaluate their prognostic value for rehabilitation outcomes. Our synthesis indicates that biomarkers reflecting distinct pathophysiological processes are emerging as key prognostic indicators. Markers of inflammation such as Tumor Necrosis Factor-alpha (TNF-α), Interleukin-6 (IL-6), and Interleukin-1 beta (IL-1β), and neuro-glial injury, including S100 Calcium-Binding Protein B (S100B), Neuron-Specific Enolase (NSE), Glial Fibrillary Acidic Protein (GFAP), and Neurofilament Light Chain (NfL), are consistently associated with poorer functional outcomes. Conversely, markers of neuroplasticity, such as Brain-Derived Neurotrophic Factor (BDNF) and Insulin-like Growth Factor-1 (IGF-1), serve as potential indicators of recovery potential, although their predictive accuracy remains inconsistent across studies. Furthermore, emerging biomarkers of synaptic activity, such as Syntaxin-1a (STX1A) and Synaptosomal-Associated Protein, 25kDa (SNAP-25), and neuromuscular junction integrity, such as C-terminal Agrin Fragment (CAF), offer novel insights into brain–periphery communication, though their clinical utility is still under investigation. While promising, the translation of these biomarkers into clinical practice is hindered by methodological limitations, including assay heterogeneity and lack of large-scale validation. Future standardization of these molecular signatures is a critical step toward implementing precision medicine in stroke rehabilitation.

## 1. Introduction

Stroke is the second leading cause of death and the third leading cause of disability worldwide, following cardiovascular and musculoskeletal disorders [1]. It substantially affects global health, with over 100 million individuals worldwide managing its long-term effects [2] and millions more affected annually [3]. While enhanced immediate medical care has led to higher survival rates following stroke, a significant proportion of survivors still struggle with persistent functional impairments long after the initial event. These ongoing deficits severely compromise their quality of life and independence during the chronic phase [4]. Predicting patient recovery remains challenging due to high variability, which limits the implementation of personalized rehabilitation programs [5]. While existing prognostic methods, such as clinical scales and brain imaging, are essential [6], they do not fully capture a patient’s underlying biological capacity for long-term functional improvement [7]. This limitation underscores the critical need for objective biological markers [8]. Circulating protein biomarkers detectable in the blood represent a promising solution, primarily because they are easily accessible, require only minimally invasive procedures, and allow for valuable longitudinal monitoring of patients [9]. These biomarkers can mirror the complex sequence of biological events triggered by a stroke, such as inflammation (e.g., Tumor Necrosis Factor-alpha [TNF-α], Interleukin-6 [IL-6], Interleukin-1 beta [IL-1β]) [10,11], neuronal and glial cell damage (e.g., Neurofilament Light Chain [NfL], Glial Fibrillary Acidic Protein [GFAP], S100 Calcium-Binding Protein B [S100B], Neuron-Specific Enolase [NSE]), alterations in synaptic function (e.g., Synaptosomal-Associated Protein, 25kDa [SNAP-25]) [12], intrinsic mechanisms mediated by growth factors (e.g., Brain-Derived Neurotrophic Factor [BDNF], Insulin-like Growth Factor-1 [IGF-1]) [13], and systemic changes, particularly in muscle tissue (e.g., C-terminal Agrin Fragment [CAF]) [14]. However, widespread clinical implementation faces practical challenges, including assay costs and the need for specialized laboratory infrastructure.

While other circulating factors, such as non-coding RNAs (e.g., microRNAs [miRNAs]), metabolomic profiles, or angiogenic factors (e.g., Vascular Endothelial Growth Factor [VEGF]), are increasingly relevant, the evidence linking them to granular functional gains remains less established and more heterogeneous compared to the proteomic markers selected for this review. In this context, we explicitly define “granular functional gains” not as general global improvements, but as specific, quantifiable recoveries in distinct domains—such as motor function (e.g., assessed by the Fugl-Meyer Assessment), activities of daily living (e.g., Functional Independence Measeure [FIM], Barthel Index), or gait parameters—as opposed to coarse measures of global disability like the modified Rankin Scale (mRS). However, unlike prior comprehensive reviews that have predominantly isolated central neuroinflammatory pathways (e.g., Iordache et al. [15]) or focused exclusively on specific neuronal injury markers like NfL (e.g., Sanchez et al. [16]), this synthesis integrates central nervous system (CNS) markers with peripheral neuromuscular signals. We posit that functional recovery depends not only on the cessation of central damage but also on the preservation of the peripheral effector apparatus. By incorporating the CAF—a specific marker of neuromuscular junction integrity—alongside established CNS markers, this review offers a genuinely new perspective: the “Brain–Muscle Axis” in rehabilitation. This approach distinguishes our work from previous syntheses by providing a holistic view of the biological substrate of recovery, bridging the gap between central command preservation and peripheral execution capacity.

**Figure 1:**
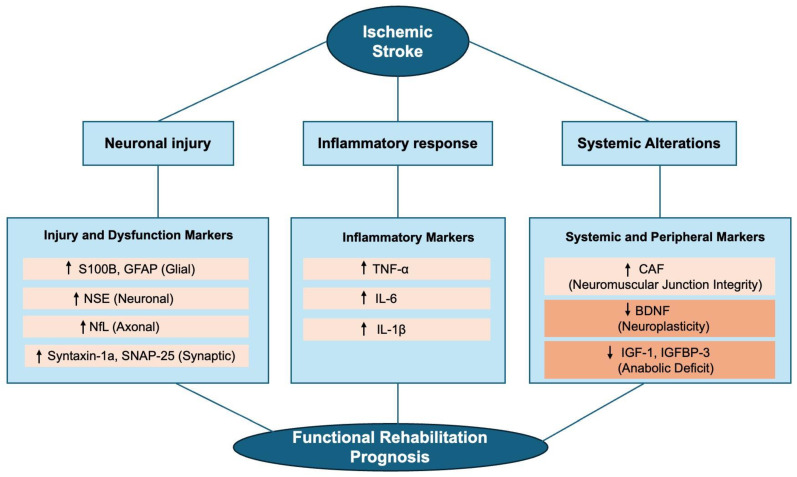
Pathophysiological cascade and biomarker signatures in post-stroke functional prognosis. The schematic illustrates the interconnected pathways triggered by ischemia—neuronal injury, systemic inflammation, and peripheral alterations—each reflected by distinct circulating biomarkers that collectively inform functional recovery potential. Symbols: ↑ indicates upregulation or increased circulating levels following stroke; ↓ indicates downregulation or decreased circulating levels. Abbreviations: S100B: S100 calcium-binding protein B; NSE: Neuron-specific enolase; GFAP: Glial fibrillary acidic protein; NfL: Neurofilament light chain; TNF-α: Tumor necrosis factor-alpha; IL: Interleukin; BDNF: Brain-derived neurotrophic factor; IGF-1: Insulin-like growth factor 1; IGFBP-3: Insulin-like growth factor binding protein 3; CAF: C-terminal agrin fragment.

A schematic representation of this comprehensive biological framework, mapping the interplay between central neurovascular injury and peripheral neuromuscular alterations, is illustrated in Figure 1. This diagram serves as a visual guide to the molecular pathways discussed throughout this review.

## 2. Search Strategy and Selection Criteria

A comprehensive literature search was conducted across PubMed, Web of Science, and Scopus to identify original research assessing the clinical utility of circulating protein biomarkers in stroke recovery and neurorehabilitation. The search included articles published up to January 2026 and was limited to the English language. The following search terms were used in various combinations: (“stroke” OR “ischemic stroke” OR “intracerebral hemorrhage”) AND (“rehabilitation” OR “functional recovery” OR “neuroplasticity” OR “motor recovery”) AND (“biomarkers” OR “circulating proteins” OR “proteomics”). Specific searches were performed for individual markers including: “Brain-derived neurotrophic factor” OR “BDNF”, “Glial fibrillary acidic protein” OR “GFAP”, “Neurofilament light chain” OR “NfL”, “S100B”, “IGFBP-3”, AND “C-terminal agrin fragment” OR “CAF”.

This study was designed as a comprehensive narrative review. Although systematic search principles were applied, a narrative synthesis format was selected over a quantitative meta-analysis. This choice was dictated by the anticipated high heterogeneity across included studies, particularly regarding (1) the variability in rehabilitation protocols; (2) the diversity of biomarker detection platforms; (3) the wide range of functional outcome measures used (global disability vs. granular motor scores). Consequently, a narrative approach provided the most appropriate framework to integrate these diverse biological and clinical findings. To ensure methodological rigor, the selection process followed a multi-step approach. Initial screening was performed based on titles and abstracts to exclude irrelevant studies and duplicate records. Subsequently, a full-text review was conducted for the remaining articles. Eligible studies included clinical trials, observational cohorts, and longitudinal studies that correlated biochemical markers with validated clinical metrics, such as the FIM, mRS, Barthel Index, or Fugl-Meyer Assessment (FMA). The final inclusion of studies for synthesis was determined based on consensus among all authors. Exclusion criteria comprised animal-only studies (unless providing essential mechanistic insights), non-English publications, and studies lacking specific correlation with functional rehabilitation outcomes. Data extraction focused on study design, biomarker temporal dynamics, detection methodology, and prognostic accuracy.

## 3. Inflammatory Cytokines

Stroke triggers a rapid onset of localized and systemic inflammatory responses, which play a critical role in ongoing brain damage. These responses are key factors in pathophysiology, as they influence both the extent of neurological deficits and the overall rehabilitation process [15]. Subsequent to stroke, complex mechanisms include the release of various cytokines and immune cells, which contribute to not only the repair process but also to an ongoing injury [17]. Systemic inflammation is a primary response following stroke and can be evaluated by measuring markedly upregulated pro-inflammatory cytokines, such as TNF-α, IL-6, and IL-1β [18]. These markers drive the inflammatory cascade and have been consistently associated with increased disability, reduced muscle strength and poorer rehabilitation outcomes [19] (Table 1).

TNF-α is a proinflammatory cytokine synthesized by two key immune cell types: macrophages [32] and T cells [33]. Along with IL-6 and IL-1β, it contributes to muscle atrophy and weakness through various mechanisms, such as the activation of nuclear factor kappa B (NF-κB) [20] and the ubiquitin–proteasome system (UPS), two pathways that are distinct, but functionally connected. NF-κB activation enhances the production of E3 ubiquitin ligases, such as MuRF-1 and Atrogin-1, which consequently drive protein breakdown via the UPS [21]. In stroke survivors, there were observed higher levels of plasma TNF-α compared to controls [22] as well as increased TNF-α mRNA expression in paretic muscles, pointing towards heightened inflammatory activity in these muscles that leads to muscle wasting and metabolic dysfunction [23]. As detailed in Table 1, these biological processes correlate with clinical indicators of disability; however, the impact of TNF-α is most consequential as a driver of catabolic decline rather than a standalone prognostic scale [24].

IL-6, another cytokine that promotes inflammation [34], is involved in various physiological and pathological processes, notably contributing in several ways to the muscle wasting seen in patients with stroke [35]. Similarly to TNF-α, a key mechanism that leads to muscle wasting involves the UPS. By enhancing ubiquitination and the breakdown of muscle regeneration factors, increased levels of IL-6 lead to reduced muscle size and function [25]. Additionally, according to research, IL-6 leads to muscle wasting through interaction with the JAK/STAT signaling pathway, especially STAT3, which is a key component of the pathway. When IL-6 binds to its receptor, it activates JAKs and subsequently phosphorylates STAT3, which disrupts muscle protein balance by stimulating catabolism and suppressing its synthesis [26]. Moreover, chronically activated STAT3 is also involved in the mitochondrial dysfunction via the increased production of mitochondrial reactive oxygen species (ROS) which negatively affects energy metabolism, ultimately contributing to muscle degeneration [27]. In stroke survivors, sustained muscle wasting can be a consequence of chronic IL-6 elevation, which promotes a catabolic state and can also lead to a shift in muscle fiber types from slow-twitch (type I) fibers to fast-twitch (type II) fibers [28].

The robust association between these cytokines and stroke severity National Institutes of Health Stroke Scale (NIHSS) or disability (mRS) is summarized in Table 1 [19]. However, despite these well-characterized molecular pathways, a significant knowledge gap remains regarding their direct correlation with granular rehabilitation metrics, such as FIM gain or gait velocity, necessitating more targeted longitudinal research.

While the majority of studies utilize the mRS to assess global outcomes [19], the impact of these cytokines on muscle catabolism implies a direct relevance to motor-specific scales. Elevated IL-6 and TNF-α levels are intrinsically linked to sarcopenia and reduced hand grip strength, key components assessed in the motor subdomains of the FIM and Trunk Control Test (TCT) [20,23,25], although direct correlations with these specific scales require further validation in large cohorts. This represents a major limitation in current stroke research; while the catabolic pathways of IL-6 and TNF-α are well characterized molecularly, the lack of granular data linking them to specific functional gains (e.g., FIM scores or gait velocity) remains a critical knowledge gap that hinders their immediate clinical utility in personalized rehabilitation.

IL-1β, another key pro-inflammatory molecule, is vital for immune responses, particularly within the central nervous system (CNS) following a stroke. When IL-1β signals to endothelial cells within the CNS, it prompts the release of granulocyte-macrophage colony-stimulating factor (GM-CSF), which subsequently converts monocytes into antigen-presenting cells, thereby intensifying neuroinflammation [29]. Furthermore, IL-1β has been shown to obstruct the migration of oligodendrocyte progenitor cells and hinder white matter repair following prolonged cerebral ischemia, which can delay functional recovery [36]. As detailed in Table 1, high IL-1β levels measured shortly after stroke correlate with markedly poorer clinical outcomes and higher scores on the NIHSS, indicating a more severe degree of neurological damage [30]. Furthermore, the predictive value of this cytokine for functional recovery at 30 days after stroke, as assessed by the mRS, underscores its role as a key mediator of the acute neuroinflammatory phase [31].

However, while the role of IL-1β in intensifying neuroinflammation through GM-CSF release is well established, its direct link to specific rehabilitation domains—such as mobility or self-care independence—remains less characterized. Most clinical evidence currently relies on global disability scales (e.g., mRS), leaving a gap in our understanding of how this cytokine specifically influences granular functional recovery during the rehabilitation phase.

Despite their prognostic value, the specificity of these cytokines for stroke-related outcomes remains a significant challenge. These markers are inherently systemic and can be upregulated by numerous non-cerebrovascular conditions, such as concurrent infections (e.g., pneumonia, urinary tract infections), autoimmune disorders, or chronic metabolic inflammation [37]. Therefore, interpreting elevated cytokine levels requires caution, as they may reflect a generalized systemic inflammatory response rather than exclusively quantifying neuroinflammation following stroke.

Beyond the systemic and local inflammatory response mediated by cytokines, the persistence of these neuroinflammatory processes often leads to direct structural compromise of the neurovascular unit. Consequently, the resulting axonal and glial damage is reflected by the release of specific injury markers into the circulation, which serve as quantifiable indicators of the extent of brain tissue loss.

## 4. Markers of Neuronal and Glial Injury

Classified as a calcium-binding protein, S100B is mainly expressed by astrocytes, the most abundant glial cells [38]. Its presence is also detected in various glial cell populations, while lower amounts can be found in specific non-neural tissues such as adipocytes, chondrocytes, and skeletal muscle satellite cells [38]. S100B has distinct functions depending on its location. Intracellularly, it contributes to the regulation of calcium homeostasis, protein phosphorylation, enzyme functions, and cytoskeleton dynamics [39]. Extracellular S100B can exert both neurotrophic and neurotoxic effects, depending on its concentration. When present in low concentrations (nanomolar), it supports neuronal growth and differentiation [40]. Studies using neuronal cell lines have shown that nanomolar concentrations of S100B enhance neurite extension and improve cell survival [41]. However, elevated concentrations can lead to neuroinflammation and programmed cell death by activating the receptor for advanced glycation endproducts (RAGE) [42]. Normally, S100B does not easily pass the blood–brain barrier (BBB) and maintains low levels in the serum [39]. Following brain damage, as in the case of stroke, when the BBB is disrupted, injured glial cells can release S100B into the bloodstream and urine [43]. As summarized in Table 2, patients who have suffered an ischemic stroke frequently show markedly higher serum S100B levels, which correlate with the extent of brain damage and less favorable prognoses [44]. The temporal dynamics of this marker, including the peak typically observed around 2–3 days (Table 2)—serve as a key indicator of functional recovery [45]. According to recent studies involving stroke patients, both initial and subsequent S100B measurements serve as indicators of functional recovery. Notably, the rate at which S100B levels decrease was identified as being linked to better neurological outcomes [46]. Regarding specific rehabilitation scales, serum S100B peaks have shown a robust inverse association with the FIM and the Barthel Index at discharge, although some studies suggest it predicts global disability more accurately than specific motor tasks [47,48]. While S100B serves as a useful biomarker for neural injury, its clinical interpretation demands careful consideration of the patient’s complete profile due to potential elevations from extracranial sources [49]. These extracranial origins, such as adipose tissue, skeletal muscle, and chondrocytes, represent a major confounder that limits the utility of S100B as a standalone stroke-specific prognostic biomarker. In multimorbid populations, systemic conditions such as obesity, recent musculoskeletal trauma, or concurrent inflammatory processes can artificially elevate serum S100B levels. This overlap makes it difficult for clinicians to discern whether high concentrations reflect persistent neuroinflammation and poor recovery potential or are merely a systemic byproduct, thereby reducing the protein’s positive predictive value for specific functional outcomes.

NSE is a glycolytic enzyme found mainly in neurons and neuroendocrine cells. Its primary function is contributing to energy metabolism by catalyzing the conversion of 2-phosphoglycerate to phosphoenolpyruvate during glycolysis [50]. Importantly, NSE is not typically secreted into the blood; however, following a stroke and subsequent neuronal damage, an initial serum peak is often observed (Table 2). This early elevation typically occurs between 7 and 18 h following stroke onset and correlates with the extent of primary neuronal injury. Subsequent complications, such as edema or increased intracranial pressure, can trigger a secondary increase in NSE levels [51]. As synthesized in Table 2, increased NSE levels at admission are linked to stroke severity, infarct volume, and poorer outcomes at three months, based on the mRS [52]. Furthermore, NSE concentrations measured within the first 24 h have demonstrated a moderate inverse correlation with the Barthel Index (Spearman’s r_s_ = −0.34, *p* = 0.001) [53], reinforcing its utility in predicting dependency in activities of daily living alongside global disability [51]. However, recent systematic reviews and prognostic updates suggest that the magnitude of this correlation varies and should be interpreted as a part of a multimodal assessment rather than a standalone marker for functional outcome [54,55]. This variability may be partially explained by disparities in sampling times; as noted in Table 2, NSE levels often peak after 24 h in many cohorts, and delayed sampling tends to yield a stronger correlation with clinical severity [54]. However, a critical limitation in the clinical utility of NSE is its lack of absolute neurospecificity. Since NSE is also abundant in erythrocytes, even minor hemolysis during blood sample collection or processing can result in false-positive elevations [56]. Consequently, rigorous pre-analytical quality control is mandatory to distinguish between true neuronal injury and artifactual increases due to sample handling [57].

GFAP, an astrocyte-specific intermediate filament protein, plays a fundamental role in maintaining cytoskeletal stability, glial cell mechanical properties, and neuronal support [58]. Under normal conditions, GFAP is not typically detected in the serum [59]; however, in patients who have experienced a brain injury such as stroke, levels often increase secondary to reactive astrogliosis—a process characterized by the proliferation and hypertrophy of astrocytes in response to damage. Despite normally exerting critical homeostatic and supportive functions in the central nervous system [60], uncontrolled astrogliosis can culminate in glial scar formation, which potentially obstructs neuronal regeneration and impedes synaptic plasticity [61]. As detailed in Table 2, serum GFAP levels rise significantly following stroke, typically reaching maximum concentration at 48 h before gradually declining [62]. Elevated concentrations are indicative of a more severe stroke and predict a poorer prognosis. This association is reinforced by recent findings where GFAP levels directly correlate with mRS scores, reflecting the patient’s degree of long-term disability and dependence [63].

Neurofilament light chain (NfL) is a structural protein of the neuronal cytoskeleton, essential for preserving axonal integrity and neuronal function [64]. It plays a crucial role in regulating nerve conduction across the central and peripheral nervous systems [64]. Under physiological conditions, low levels of NfL are continuously released from neurons into the systemic circulation, reflecting normal neuroaxonal turnover and remodeling [65]. As illustrated in Table 2, stroke triggers a marked rise in blood concentrations, generally peaking around the 7th day [16], with the magnitude of this elevation correlating with the extent of neuronal damage [66]. Subsequently, levels decrease slowly, persisting above baseline for 3–6 months or longer [67]. Consequently, serial measurements offer valuable insights into the recovery trajectory [68]. In the chronic phase after stroke, elevated levels are associated with poorer functional improvement, particularly in domains related to gait, balance, and cognition [66]. Specifically, recent studies have highlighted the prognostic value of NfL for locomotor recovery, demonstrating a robust and clinically relevant association between elevated plasma levels and lower scores on the Functional Ambulation Categories (FAC). This suggests that axonal loss directly impacts the corticospinal pathways required for independent walking and dynamic balance [69]. Regarding global disability, Table 2 summarizes the consistent correlations found between high NfL levels and poorer outcomes across standard metrics. High NfL concentrations correspond to greater functional impairment on the mRS [70] and greater neurological deficits on the NIHSS [71]. Similarly, negative associations with the Barthel Index underscore the link between axonal injury and increased dependence in activities of daily living [72].

While injury markers quantify the extent of structural loss, the recovery process depends equally on the functional integrity of the remaining neural networks, particularly at the level of synaptic transmission.

**Table 2:** Markers of Neuronal and Glial Injury and Their Correlation with Stroke Rehabilitation Outcomes.

Biomarker	Stroke Phase (Peak Time)	Key Findings	Clinical Assessment Scales	Effects on Outcome	References
S100B	Acute (Peak 2–3 days)	Higher levels correlate with extensive brain damage. Rate of decline predicts better recovery.	mRS, Barthel Index, FIM	↑ Levels = ↓ Recovery	[44,46]
NSE	Acute (7–18 h)	Linked to infarct volume and worse functional outcomes at 3 months.	mRS, NIHSS, Barthel Index	↑ Levels = ↓ Recovery	[50,52,53]
GFAP	Acute (Peak 48 h)	Increased concentrations suggest severe stroke and predict greater functional disability.	mRS, NIHSS	↑ Levels = ↓ Recovery	[62,63]
NfL	Subacute (~7 days)	Reflects axonal degeneration; associated with deficits in gait (FAC), balance, and cognition.	mRS, NIHSS, Barthel Index, FAC	↑ Levels = ↓ Recovery	[16,66,69,70,71,72]

Symbols: ↑ indicates increased levels; ↓ indicates poor recovery. Abbreviations: FAC: Functional Ambulation Categories; FIM: Functional Independence Measure; GFAP: Glial Fibrillary Acidic Protein; mRS: Modified Rankin Scale; NfL: Neurofilament Light Chain; NIHSS: National Institutes of Health Stroke Scale; NSE: Neuron-Specific Enolase; S100B: S100 calcium-binding protein B.

## 5. Markers Related to Synaptic Function

Syntaxin-1a (STX1A) and Synaptosomal-Associated Protein, 25kDa (SNAP-25) are integral components of the Soluble N-ethylmaleimide-sensitive factor Attachment Protein Receptor (SNARE) complex, playing a pivotal role in neurotransmitter release at presynaptic terminals [73,74]. STX1A facilitates exocytosis by interacting with other SNARE proteins, such as synaptobrevin (VAMP2) and SNAP-25, to mediate the docking and fusion of synaptic vesicles with the presynaptic membrane [75]. Beyond its structural role, SNAP-25 demonstrates a protective function following ischemic stroke. Elevated bloodstream levels post event are associated with the inhibition of ferroptosis—a form of programmed cell death—thereby reducing ischemic injury and supporting neural survival and functional recovery [76].

Following ischemic stroke, the disruption of the BBB allows these synaptic proteins to leak into the systemic circulation. Researchers have detected elevated levels across different blood fractions, including serum, peripheral blood mononuclear cells (PBMCs), and neuronal-derived extracellular vesicles (NDEs). As summarized in Table 3, recent clinical evidence establishes a robust link between STX1A concentrations and validated outcomes: specifically, the NIHSS for initial severity and the Modified Barthel Index (MBI) for functional status. This association demonstrates that these proteins provide a unique quantitative measure of synaptic integrity that traditional markers of necrosis fail to capture. Beyond reflecting the acute extent of the brain injury, STX1A and SNAP-25 serve as indicators of fundamental neurophysiological alterations involving both synaptic damage and subsequent repair activities [77].

A critical question remains regarding how these markers, which leak into the serum due to synaptic disintegration, can be linked to neuroprotective mechanisms. This apparent paradox may be explained by the temporal dynamics of recovery. In the hyper-acute phase, elevated serum STX1A and SNAP-25 likely result from passive release due to necrotic damage, thus correlating with stroke severity (NIHSS). However, in the subacute phase, the brain attempts to restore connectivity through active synaptogenesis and remodeling [86]. We hypothesize that elevated levels during this window might also reflect a ‘spillover’ from the compensatory upregulation of synaptic machinery needed for plasticity. Therefore, these biomarkers might serve a dual role: indicators of structural loss in the acute phase, and essential biological evidence of active synaptic remodeling efforts during the rehabilitation phase. This dual functionality justifies their clinical necessity in monitoring the transition from neural injury to functional recovery.

The maintenance and repair of these synaptic connections are not autonomous processes; they are heavily dependent on the presence of specific growth factors that provide the necessary anabolic support for tissue regeneration.

## 6. Growth Factors

IGF-1 is a peptide hormone produced predominantly by the liver in response to growth hormone (GH) stimulation [87]. Acting as both a growth factor [88] and a neurotrophin [89], this anabolic protein is crucial for muscle functionality, neuron survival, and facilitating synapse formation [78,90,91]. At the molecular level, IGF-1 binding to its receptor (IGF-1R) triggers the PI3K/Akt/mTOR signaling axis. This pathway is pivotal for inhibiting apoptotic cascades, stimulating protein synthesis, and promoting both axonal regeneration and skeletal muscle hypertrophy during the recovery phase. Specifically, this cascade shifts the metabolic balance towards anabolism by simultaneously stimulating protein synthesis and suppressing the expression of atrophy-related E3 ubiquitin ligases (MuRF1 and Atrogin-1) [79,92], a mechanism essential for counteracting post-stroke sarcopenia. The activity and availability of IGF-1 are tightly modulated by insulin-like growth factor binding proteins (IGFBPs), with IGFBP-3 being the most abundant circulating carrier [93]. IGFBP-3 exerts a dual regulatory role: by binding and isolating IGF-1, IGFBP-3 effectively obstructs its interaction with IGF-1R, thereby suppressing its anabolic actions; conversely, it may also increase the local concentration of IGF-1 in proximity to its receptor, thus potentiating its action [94]. Beyond binding IGF-1, IGFBP-3 also exerts independent functions by interacting with cell surface receptors [95] and nuclear proteins [96] to modulate apoptosis and cell survival, contributing to myogenesis [94]. Evidence indicates a synergistic interaction where IGF-1 promotes IGFBP-3 release, which subsequently facilitates myoblast differentiation—a foundational step in building muscle tissue [82].

As highlighted in Table 3, reduced serum levels of IGF-1 and IGFBP-3 are characteristic of the post-stroke environment [97]. Specific cohorts have demonstrated a substantial reduction of 25% in IGF-1 levels and a 40% reduction in IGFBP-3 levels in stroke survivors, compared to healthy controls, indicating a severe deficit in anabolic signaling. These diminished profiles correlate with the clinical outcomes summarized in Table 3, specifically declines in muscle mass, impaired neuromuscular function and atrophy in key muscles such as the quadriceps and hamstrings [98].

By stimulating cellular growth and protein synthesis, these factors lay the groundwork for the more complex structural adaptations and long-term potentiation that define neuroplasticity.

## 7. Markers of Neuroplasticity

BDNF functions as a pivotal neurotrophin, governing essential processes such as neuronal survival, synaptic remodeling and neurogenesis [99]. In stroke rehabilitation, BDNF is recognized as a fundamental molecular driver of neuroplasticity, facilitating the neural adaptations required for motor and functional recovery [100]. Crucially, unlike biomarkers that passively reflect tissue damage (e.g., NfL or S100B), BDNF is an active driver of recovery, facilitating long-term potentiation—which is the cellular foundation of learning and memory formation during rehabilitation [83].

Mechanistically, BDNF exerts its proneurogenic effects primarily by binding to the high-affinity Tropomyosin receptor kinase B (TrkB). This interaction activates downstream signaling cascades, including the MAPK/ERK and PI3K/Akt pathways, which are essential for long-term potentiation, dendritic branching, and neuronal survival [101,102].

As summarized in Table 3, circulating levels of BDNF are substantially altered following stroke. Consistently, research indicates that patients across both the acute and chronic phases of stroke present with markedly reduced serum and plasma BDNF levels compared to healthy individuals [84,103,104]. These lower levels have been correlated with greater stroke severity and poorer long-term functional outcomes [105]. In contrast, higher baseline levels of BDNF are associated with better functional independence and a reduced incidence of post-stroke depression, a common comorbidity that negatively impacts rehabilitation efficacy [106].

Uniquely among these biomarkers, BDNF is modulated by physical activity. Systematic reviews and meta-analyses in stroke survivors show that both single sessions and programs of high-intensity aerobic exercise markedly increase circulating BDNF concentrations, thereby creating a permissive environment for neuroplasticity [107,108].

However, genetic variability markedly influences this response. Specifically, the Val66Met polymorphism, found in approximately 30% of the population, impairs activity-dependent BDNF secretion. This genetic variation has been linked to poorer motor recovery and may alter the clinical efficacy of physical training [109]. While animal models consistently show that this polymorphism limits the exercise-induced rise in BDNF and its neuroprotective benefits [85], the picture in humans is more complex. Although many studies suggest that carriers of the ’Met’ allele fail to upregulate BDNF as effectively as ’Val/Val’ homozygotes following aerobic or high-intensity exercise, others report inconsistent findings, suggesting that human outcomes are likely influenced by additional variables such as age and sex [110,111]. Despite this heterogeneity, the actionable takeaway for clinicians is that Val66Met genotyping identifies a specific subgroup of patients who may require ‘higher doses’ or more prolonged sessions of aerobic exercise to trigger the same neuroplastic response as Val/Val carriers. Rather than diluting its applicability, this complexity underscores the need for personalized rehabilitation protocols where exercise intensity is titrated based on genetic susceptibility to optimize BDNF-mediated recovery.

While multiple studies confirm that low acute-phase BDNF serves as a robust independent predictor of global disability, showing a linear relationship with mRS scores up to 7 years following stroke [105], modern neurorehabilitation demands more granular assessment metrics. Consequently, research has expanded to investigate the relationship between BDNF and specific functional scales. For instance, serum BDNF levels measured at admission have shown a statistically significant, albeit weak, correlation with the FIM motor subscores at discharge (r = 0.173), suggesting a potential link to motor relearning capacity [112]. Furthermore, genetic studies provide deeper insight; carriers of the BDNF Met allele, characterized by lower secretion rates, exhibit consistently poorer outcomes on the FIM cognitive subscale, particularly in domains of expression and problem-solving, compared to Val/Val carriers [113].

Critically, however, the clinical utility of BDNF is nuanced. A major limitation that must be emphasized is its low diagnostic accuracy (AUC ~ 0.58) for predicting specific motor improvements. This weakness underscores that BDNF should not be used as a sole predictor, despite its correlations with the FIM motor subscores [112]. Consequently, its true clinical utility lies in its role as a dynamic indicator of ‘rehabilitation readiness’. When integrated into a multimodal assessment, BDNF serves as a robust biological marker for monitoring the efficacy of high-intensity interventions [107]. This is supported by evidence that elevated BDNF levels at 3 months are linked to markedly reduced risks of long-term disability and mortality [114]. Similarly, in the chronic phase, while reduced BDNF levels do not correlate strongly with the Stroke Specific Quality of Life Scale (SS-QOL) [115], they remain a key indicator of global recovery potential. Therefore, BDNF should be interpreted as a foundational tool alongside clinical scales to validate the brain’s response to neurorehabilitative stimuli, rather than as a standalone predictor of individual trajectories.

**Figure 2:**
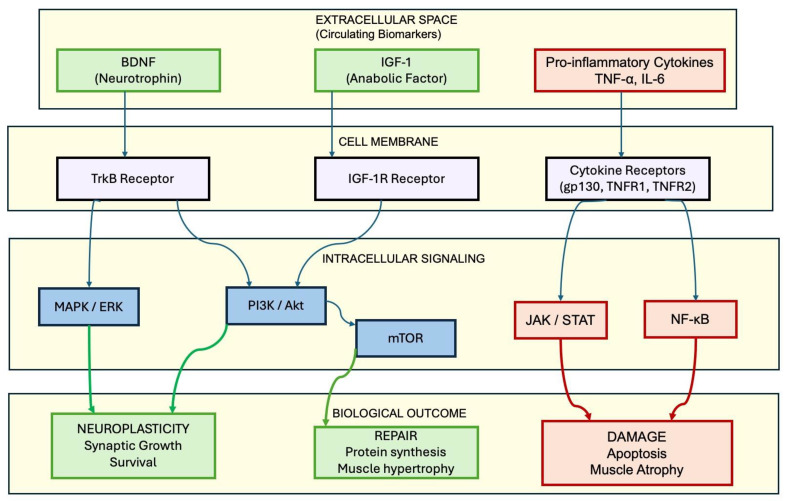
Schematic representation of molecular signaling pathways in stroke recovery. The diagram contrasts neurorestorative mechanisms (left and center panels) involving BDNF/TrkB and IGF-1/PI3K axes that promote plasticity and hypertrophy, with pro-inflammatory pathways (right panel) that drive apoptosis and muscle atrophy. Abbreviations: TrkB: Tropomyosin receptor kinase B; mTOR: Mammalian target of rapamycin; JAK/STAT: Janus kinase/signal transducer and activator of transcription; NF-κB: Nuclear factor kappa B.

While each of these biomarkers provides distinct prognostic value, their clinical significance is ultimately determined by the complex interplay between the molecular cascades they trigger. This antagonistic relationship between neurorestorative signaling and pro-inflammatory pathways is schematically synthesized in Figure 2, highlighting the biological substrate that governs functional recovery trajectories.

## 8. Markers of Neuromuscular Junction Integrity

CAF is a specific protein fragment, produced when neurotrypsin cleaves agrin, a large proteoglycan molecule essential for preserving the structure and function of the neuromuscular junctions (NMJs) [116]. Agrin, which is released by motor neurons, binds to the muscle-specific kinase (MuSK) receptor on the postsynaptic membrane. This binding triggers a signaling cascade resulting in the phosphorylation and aggregation of acetylcholine receptors (AChRs) at the NMJ [117], a step essential for efficient neuromuscular transmission [118]. Following stroke, particularly of ischemic origin, a cascade of pathological processes occurs within the neural and muscular systems. A key mechanism involves increased proteolytic activity driven by the enzyme neurotrypsin, which plays a critical role in agrin’s cleavage [80]. Consequently, the formed CAF enters the circulation and can be quantified in the serum [81]. The recognition of this fragment as a notable biomarker that indicates damage of the NMJ is continuously growing due to its association with muscle wasting in several conditions [88,89]. While multiple isoforms of CAF exist, but CAF22 is the sub-fragment typically analyzed as a circulating biomarker for muscle atrophy and NMJ integrity [119].

As highlighted in Table 3, markedly elevated serum CAF22 levels have been observed in patients with stroke upon admission, compared to healthy controls. Although these levels partially decreased by the time of discharge, they still remained higher than those in healthy individuals [88]. Therefore, in stroke survivors, increased levels of CAF22 are clinically relevant as they can be correlated with muscle mass and function [89]. Specifically, regarding functional assessment, serum CAF levels have demonstrated a robust inverse correlation with handgrip strength, a validated surrogate measure for global motor recovery often used alongside the FIM. Furthermore, elevated CAF levels in the subacute phase have been associated with lower scores on the Barthel Index, highlighting its potential to predict dependency in activities of daily living [88].

## 9. Clinical Utility and Future Directions in Biomarker-Guided Rehabilitation

Integrating these biomarkers into clinical practice holds the potential to redefine neurorehabilitation, facilitating a transition from generic protocols to precision medicine. Synthesizing the evidence reviewed, we hypothesize three critical avenues for biomarker-guided strategies, supported by emerging consensus on stroke recovery frameworks [120].

Patient Stratification and Resource Allocation. Patients presenting with high acute levels of NfL or GFAP (indicating severe structural damage) combined with low baseline BDNF (indicating poor neuroplastic reserve) could be identified early as poor responders. Moving beyond theoretical models, recent pilot studies have already begun leveraging such integrated biological and clinical profiles to predict ‘upper limb recovery potential’, allowing for more targeted allocation of intensive robotic-assisted training [121]. These patients might require intensified rehabilitation protocols (e.g., robotic-assisted training, non-invasive brain stimulation) or longer durations of therapy compared to those with favorable biomarker profiles. Conversely, patients with high inflammatory load (IL-6, TNF-α) might benefit from early immunomodulatory interventions alongside physical therapy to prevent the transition to chronic sarcopenia.

Monitoring Therapeutic Efficacy. Currently, rehabilitation efficacy is measured primarily by functional scales, which can be subjective and slow to change. Serum biomarkers, particularly BDNF and IGF-1, could serve as dynamic indicators of training intensity. While IGF-1 has shown limited utility as a dynamic marker in chronic stroke due to inconsistent changes following exercise [122], BDNF is more closely linked to acute neurobiological responses [107,123,124]. Meta-analytic data show that high-intensity aerobic training, specifically high-intensity interval training (HIIT) and (sprint interval training) SIT protocols, can reliably increase serum BDNF in stroke survivors. This dose–response relationship supports the use of serum BDNF as a real-time feedback tool to titrate exercise intensity [107,125,126]. Recent trials are investigating the use of such molecular signatures to identify ‘early responders’ to specific interventions like repetitive transcranial magnetic stimulation and high-intensity aerobic training [127]. Since aerobic exercise is known to upregulate BDNF, failing to see a rise in serum BDNF after 2–3 weeks of rehabilitation could prompt clinicians to modify the exercise intensity, type, or duration for that specific patient. This lack of response is clinically consequential and may indicate suboptimal intensity, genetic resistance (e.g., Val66Met polymorphism), or blunted responsiveness due to comorbid depression [84,112]. In such cases, the literature supports a strategic shift: increasing exercise intensity (progressing to HIIT or SIT), switching to interval modalities, or extending intervention duration [125,126]. Furthermore, because serum BDNF peaks immediately post exercise and returns to baseline within 30–90 min [120], the timing of measurement is critical. Ultimately, serum BDNF should be interpreted within a multimodal framework: integrating molecular, imaging and computational biomarkers to enable true precision rehabilitation and individualized therapy [128,129].

Targeting Neuromuscular Integrity. The identification of elevated CAF levels in the subacute phase signals active neuromuscular junction degeneration. This warrants specific interventions focused on high-resistance strength training or electrical stimulation to counteract sarcopenia, rather than generic aerobic conditioning alone.

Conversely, it is crucial to acknowledge that certain well-established biomarkers have shown inconsistent correlations with functional recovery outcomes. For instance, C-reactive protein (CRP), despite being a robust predictor of mortality and recurrent vascular events, often lacks the specificity required to predict granular motor gains in rehabilitation settings compared to cytokines like IL-6 [130,131]. Similarly, while Matrix Metalloproteinase-9 (MMP-9) is strongly associated with acute BBB disruption and hemorrhagic transformation, its correlation with long-term functional independence measures (e.g., Barthel Index, mRS) remains contradictory across studies, suggesting it may be less suitable as a standalone rehabilitation marker [132,133,134].

As most studies are observational, future clinical trials should move beyond correlation. The transition from experimental correlation to routine clinical stratification requires further validation in large-scale multicenter trials to establish standardized ‘threshold values’ for decision-making [135]. We need biomarker-guided interventional trials where patients are randomized to specific rehabilitation arms based on their biological profile (e.g., high inflammation versus slow neuroplasticity groups) to see if targeted strategies improve outcomes better than standard care.

Beyond their prognostic role, a critical step toward clinical translation is the integration of these markers into rehabilitation trials as active determinants of study design, specifically as stratification tools and surrogate endpoints. As stratification tools, baseline biomarker profiles could identify ‘biological non-responders’ to standard therapy. For instance, patients with low biological reserves (e.g., suppressed BDNF) might be stratified into intensified protocols or ‘priming’ interventions (such as aerobic exercise or non-invasive brain stimulation) prior to motor training to boost neuroplasticity. Conversely, patients with high systemic inflammation (elevated IL-6) might require immunomodulatory adjuncts to facilitate recovery. As surrogate endpoints, fluid biomarkers offer objective, immediate readouts of therapeutic efficacy, overcoming the latency and subjectivity of functional scales. For example, a reduction in serum NfL levels could serve as an early, quantifiable indicator of neuroaxonal preservation, allowing for rapid ‘go/no-go’ decisions in Phase II trials of neurorestorative therapies before long-term functional gains become clinically apparent.

However, a significant hurdle in the clinical translation and large-scale validation of these hypotheses is the variability in detection methods. For markers such as NfL and BDNF, the shift from traditional Enzyme-Linked Immunosorbent Assay (ELISA) to ultra-sensitive Single Molecule Array (Simoa) technology has led to variable absolute concentrations and reference ranges [136,137]. This variability, driven by differences in analytical sensitivity and assay design, means that while platforms like Roche Elecsys and Simoa show strong correlation, they yield different absolute values, necessitating platform-specific clinical cut-offs [138]. Furthermore, systematic biases and the current lack of SI-traceable calibrators complicate direct cross-study comparisons [139]. Until global standardization and harmonization are achieved, clinicians must interpret biomarker results strictly within the context of the specific technology employed, a factor that remains a major challenge for the universal implementation of biomarker-guided rehabilitation.

**Figure 3:**
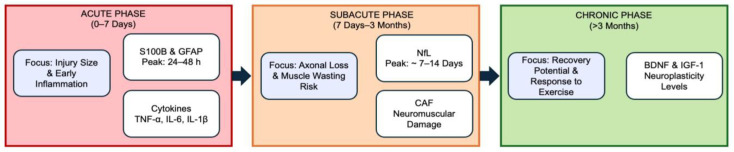
Temporal timeline of representative biomarker utility in stroke rehabilitation. The diagram illustrates the integrated biological trajectory of stroke recovery divided into three prognostic windows, highlighting the shift from acute injury markers to chronic neuroplasticity indicators. Abbreviations: S100B: S100 calcium-binding protein B; GFAP: Glial fibrillary acidic protein; TNF-α: Tumor necrosis factor-alpha; IL: Interleukin; NfL: Neurofilament light chain; CAF: C-terminal agrin fragment; BDNF: Brain-derived neurotrophic factor; IGF-1: Insulin-like growth factor 1.

To synthesize these clinical applications and provide a unified framework for the practitioner, Figure 3 illustrates the integrated biological trajectory of stroke recovery, mapping the critical prognostic windows. Complementing this visual timeline, Table 4 summarizes the biological dynamics, optimal measurement windows, and specific prognostic value for each biomarker across the different phases of stroke recovery.

## 10. Limitations and Future Directions: Bridging the Gap to Clinical Utility

While the potential of circulating proteins to revolutionize stroke rehabilitation is evident, several critical methodological limitations currently impede their routine clinical implementation. This review deliberately focused exclusively on protein biomarkers. We acknowledge that non-coding RNAs (miRNAs) and metabolomics represent a rapidly evolving field that warrants separate investigation, as recent studies suggest they may offer distinct advantages in detecting early cellular responses to ischemic injury. These approaches provide a complementary biological layer, reflecting epigenetic and metabolic shifts that proteomic markers alone may not capture [140,141].

A primary challenge, as highlighted by the variability in the current literature, lies in the heterogeneity of assay protocols across studies. Differences in detection platforms (e.g., conventional ELISA versus ultra-sensitive Simoa), variability in sample handling, and the lack of standardized cut-off values make it difficult to compare results across cohorts and establish universal reference ranges. To address these hurdles, concrete steps toward harmonization are being advocated by international efforts such as the International Federation of Clinical Chemistry (IFCC) and the NIH Biomarkers Consortium. These initiatives aim to develop standardized reference materials and unified analytical protocols to ensure that measurements, particularly for markers like NfL and BDNF, remain consistent across different laboratory settings [142].

Furthermore, the temporal dynamics of these biomarkers exhibit substantial heterogeneity. As noted in this review, proteins like GFAP peak early (24–48 h), while NfL levels rise gradually and remain elevated for months. Most existing studies rely on static assessments at single time-points, which may fail to capture the complex, longitudinal biological trajectory of recovery. Additionally, the influence of comorbidities must be considered, as biomarker levels are often confounded by systemic conditions. For instance, renal failure can substantially compromise NfL clearance, leading to elevated levels that reflect decreased glomerular filtration rather than ongoing neuroaxonal damage, while systemic inflammation from concurrent infections can mask stroke-specific inflammatory profiles (e.g., IL-6, TNF-α). These confounding factors complicate the differentiation between biological signals of injury and signals of repair. Consequently, future research must distinguish between markers that simply predict a poor baseline (severity markers) and those that predict the biological capacity for improvement (true rehabilitation markers). Linked to this challenge is the discrepancy between mechanistic knowledge and clinical correlation; while the pathways for inflammation and atrophy are molecularly well characterized, there is a paucity of evidence correlating biomarker levels with specific, granular rehabilitation metrics, such as gait speed or fine motor recovery scores.

Consequently, findings regarding prognostic accuracy often lack consistency, particularly for emerging markers of synaptic integrity (STX1A, SNAP-25) where large-scale validation in diverse stroke populations remains limited.

To overcome these evidentiary gaps, future investigations must prioritize several key directions:

- Standardization: To move from research to practice, participation in initiatives like the Foundation for the National Institutes of Health (FNIH) Biomarkers Consortium is essential to establish standardized assay protocols and reference ranges, ensuring analytical precision and reproducibility. Furthermore, the adoption of the ‘Biomarkers, EndpointS and other Tools’ (BEST) framework, developed by the FDA and NIH, is crucial. The BEST resource provides standardized terminology and a systematic path for validating biomarkers as qualified clinical tools, ensuring they meet the rigorous regulatory standards required for guiding rehabilitation interventions [143,144].
- Longitudinal design: Conducting adequately powered longitudinal cohort studies that measure biomarker dynamics at multiple time-points during the rehabilitation phase, correlating these fluctuations directly with specific therapeutic responses rather than static outcomes.
- Multi-Marker Panels: Integrating inflammatory, structural, and neuroplasticity markers into a single predictive score could substantially bolster prognostic sensitivity and specificity compared to individual biomarkers alone.

Another critical limitation in the field lies in the heterogeneity of rehabilitation protocols. Unlike pharmacological treatments, physical rehabilitation often lacks standardized guidelines regarding the specific type, frequency, and intensity of exercises, leading to variability in clinical outcomes. Currently, there is a scarcity of rehabilitation trials that utilize biomarkers as primary endpoints to validate these interventions. Therefore, future studies should prioritize integrating biomarkers of CNS injury (e.g., NfL, GFAP) and inflammation into randomized controlled trials. This integration is essential to scientifically verify current rehabilitation practices, establishing a biological basis for determining the optimal timing and intensity of therapy.

Finally, regarding the review process itself, we acknowledge that this study was conducted without external funding. While we maintained rigorous methodological standards, the absence of dedicated financial resources is a factor that may limit access to certain high-cost proprietary databases and specialized systematic review tools, a common constraint in independent academic research.

## 11. Conclusions

Due to pronounced inter-individual variability, accurately predicting functional recovery after a stroke remains a difficult task. This review assessed the potential of circulating proteins to fill this predictive gap. The evidence indicates distinct prognostic patterns: biomarkers of inflammation (e.g., TNF-α, IL-6, IL-1β) and neuronal or glial damage (e.g., S100B, GFAP, NSE, NfL) tend to correlate with less favorable outcomes, especially when measured in the acute phase of a stroke. In contrast, markers of neuroplasticity and repair (such as BDNF, IGF-1 and IGFBP-3) appear to be protective, with higher levels being associated with better recovery.

Moving forward, we propose a specific, testable model for future studies based on longitudinal multimarker panels. Specifically, synaptic (STX1A, SNAP-25) and neuromuscular junction (CAF) markers should be integrated into comprehensive biological profiles to bridge the gap between neural repair and muscular integrity. To advance from discovery to clinical utility, research must transition toward standardized protocols that correlate these biological data not only with global disability scores (e.g., mRS) but also with granular functional outcomes (e.g., Fugl-Meyer Assessment, sarcopenia indices). Ultimately, the implementation of biomarker-guided algorithms—is essential for moving stroke recovery from a ‘one-size-fits-all’ approach toward a model of precision neurorehabilitation.

## Figures and Tables

**Table 1:** Summary of pro-Inflammatory Cytokines (TNF-α, IL-6, IL-1β) and their correlation with clinical prognostic scales in stroke rehabilitation.

Biomarker	Stroke Phase (Peak Time)	Key Findings	Clinical Assessment Scales	Effects on Outcome	References
TNF-α	Acute (<24 h)	Activates NF-κB and UPS; contributes to muscle atrophy and weakness.	mRS, NIHSS, FIM	↑ Levels = ↓ Recovery	[20,21,22,23,24]
IL-6	Acute to Chronic	Promotes chronic catabolic state (sarcopenia); correlates with stroke severity and disability.	mRS, NIHSS, Handgrip Strength	↑ Levels = ↓ Recovery	[19,25,26,27,28]
IL-1β	Acute	Intensifies neuroinflammation; high acute levels correlate with infarct volume and worse functional scores.	mRS, NIHSS	↑ Levels = ↓ Recovery	[29,30,31]

Symbols: ↑ indicates increased levels; ↓ indicates poor recovery. Abbreviations: FIM: Functional Independence Measure; IL-1β: Interleukin-1β; IL-6: Interleukin-6; mRS: Modified Rankin Scale; NF-κB: Nuclear Factor Kappa B; NIHSS: National Institutes of Health Stroke Scale; TNF-α: Tumor Necrosis Factor-alpha, UPS ubiquitin–proteasome system.

**Table 3:** Different Biomarkers and Their Correlation with Stroke Rehabilitation Outcomes.

Biomarker	Category	Key Findings	Stroke Phase (Peak Time)	Clinical Assessment Scales	Effects on Outcome	References
STX1A	Synaptic	Reflects synaptic damage and repair processes; predicts clinical outcome.	Acute	NIHSS, Barthel Index	↑ Levels = Severity	[77]
SNAP-25	Synaptic	Inhibits ferroptosis, potentially supporting neural survival despite injury and predicts functional recovery.	Acute	NIHSS, Barthel Index	↑ Levels = Protection	[76,77]
IGF-1 & IGFBP-3	Growth factors	Deficit leads to anabolic failure, muscle atrophy, and weakness.	Chronic	Muscle Strength, mRS	↓ Levels = ↓ Function	[78,79]
CAF/CAF22	NMJ integrity	Indicates NMJ damage; correlated with muscle wasting and poorer physical prognosis.	Subacute	Handgrip Strength, Barthel Index	↓ Levels = ↓ Outcomes	[80,81]
BDNF	Neuroplasticity	Key driver of neuroplasticity. Low acute levels predict disability; levels rise with aerobic exercise.	Acute (Prognostic) Chronic (Monitoring)	Acute: mRS, FIM Chronic: FIM (Gain), FAC	Acute: ↓ Levels = ↓ Outcomes Chronic: ↑ with Exercise	[82,83,84,85]

Symbols: ↑ indicates increased levels or upregulation; ↓ indicates decreased levels or downregulation. Abbreviations: BDNF: Brain-Derived Neurotrophic Factor; CAF: C-Terminal Agrin Fragment; FAC: Functional Ambulation Categories; FIM: Functional Independence Measure; IGF-1: Insulin-like Growth Factor-1; IGFBP-3: Insulin-like Growth Factor Binding Protein-3; mRS: Modified Rankin Scale; NIHSS: National Institutes of Health Stroke Scale; NMJ: Neuromuscular Junction; SNAP-25: Synaptosomal-Associated Protein, 25kDa; STX1A: Syntaxin-1a.

**Table 4:** Unified synthesis of biomarker temporal dynamics and clinical prognostic windows in stroke rehabilitation.

Biomarker	Biological Source	Peak Time	Prognostic Window	Clinical Utility in Rehabilitation	References
S100B/NSE	Glial/Neuronal	24–72 h	Acute (<72 h)	Early prediction of global functional independence (FIM/mRS scores) and infarct size.	[36,37,41,42,43,44,45,46,48,49,50,51]
GFAP	Astrocytes	24–48 h	Acute (<72 h)	Assessment of reactive astrogliosis; predictor of early neurological deterioration.	[58,59,62,63]
NfL	Axons	~7 days (stable for months)	Subacute (7 days–3 months)	Long-term monitoring neuroaxonal loss; correlates with gait speed and chronic disability.	[16,64,65,69,70,72]
IL-6/TNF-α	Immune Cells	0–24 h (can remain high)	Acute to Subacute	Markers of systemic inflammation; predictive of post-stroke muscle wasting and poor recovery.	[18,19,20,23,28,29,35,36]
BDNF	Neurons	Highly variable	Chronic (>3 months)	Indicators of “rehabilitation readiness”; monitors neuroplasticity response to physical exercise.	[82,84,85,92,93,102]
CAF	NMJ	Subacute stage	Subacute to Chronic	Specific marker for neuromuscular junction integrity and risk of post-stroke sarcopenia.	[80,81,114,119]

This table provides a unified model of the biomarkers discussed in this review. The “Prognostic Window” represents the optimal periods for serum measurement to achieve maximum predictive value for rehabilitation outcomes. “Peak times” refer to expected maximum concentration levels in peripheral blood following stroke onset. Abbreviations: BDNF: Brain-Derived Neurotrophic Factor; CAF: C-Terminal Agrin Fragment; FIM: Functional Independence Measure; GFAP: Glial Fibrillary Acidic Protein; IL-6: Interleukin-6; mRS: Modified Rankin Scale; NfL: Neurofilament Light Chain; NMJ: Neuromuscular Junction; NSE: Neuron-Specific Enolase; S100B: S100 Calcium-Binding Protein B; TNF-α: Tumor Necrosis Factor-Alpha.

## Data Availability

No new data were created or analyzed in this study. Data sharing is not applicable to this article.

## Abbreviations

The following abbreviations are used in this manuscript:

AChRs	Acetylcholine Receptors
BBB	Blood–Brain Barrier
BDNF	Brain-Derived Neurotrophic Factor
CAF	C-Terminal Agrin Fragment
FAC	Functional Ambulation Categories
FIM	Functional Independence Measure
FNIH	Foundation for the National Institutes of Health
GFAP	Glial Fibrillary Acidic Protein
GH	Growth Hormone
GM-CSF	Granulocyte-Macrophage Colony-Stimulating Factor
HIIT	High-Intensity Interval Training
IGF-1	Insulin-like growth factor-1
IGF-1R	Insulin-like growth factor-1 receptor
IGFBPs	Insulin-like growth factor binding proteins
IGFBP-3	IGF binding protein 3
IL-1β	Interleukin-1β
IL-6	Interleukin-6
JAK/STAT	Janus kinase/signal transducer and activator of transcription
mRS	Modified Rankin Scale
mTOR	Mammalian target of rapamycin
MuSK	Muscle-specific receptor tyrosine kinase
NDEs	Neuronal-derived extracellular vesicles
NF-κB	Nuclear factor kappa B
NfL	Neurofilament light chain
NIHSS	National Institutes of Health Stroke Scale
NMJs	Neuromuscular Junctions
NSE	Neuron-Specific Enolase
PBMCs	Peripheral Blood Mononuclear Cells
PI3K/Akt:	Phosphoinositide 3-kinase/protein kinase B
RAGE	Receptor for Advanced Glycation Endproducts
ROS	Reactive Oxygen Species
SIT	Sprint Interval Training
SNARE	Soluble N-ethylmaleimide-sensitive factor Attachment Protein Receptor
SNAP-25	Synaptosomal-Associated Protein, 25 kDa
STX1A	Syntaxin-1a
TNF-α	Tumor Necrosis Factor-α
UPS	Ubiquitin–Proteasome System
